# In this issue: Volume 114, Issue 11, November 2023

**DOI:** 10.1111/cas.16002

**Published:** 2023-11-10

## Combination of PARP inhibitor and CDK4/6 inhibitor modulates cGAS/STING‐dependent therapy‐induced senescence and provides “one‐two punch” opportunity with anti‐PD‐L1 therapy in colorectal cancer


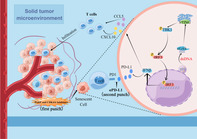


Poly(ADP‐ribose) polymerase (PARP) is a family of proteins that regulates cellular processes such as DNA repair, genomic stability, and cell death pathways. PARP inhibitors (PARPi) have demonstrated remarkably lethal effects on tumor cells that lack DNA repair pathways, particularly cells with BRCA1/2 mutations. While they hold significant promise in the treatment of cancer cells harboring these mutations, their efficacy in colorectal cancer (CRC) is limited due to the low frequency of BRCA1/2 mutations.

Nevertheless, combining PARPi with other therapeutic agents could potentially broaden their applications in the treatment of CRC. To test this hypothesis, Wang et al conducted a series of experiments using in vitro and in vivo CRC models. Their aim was to examine the combined effect of the PARPi – ‘talazoparib’, and cell‐cycle dependent kinase‐4/6 inhibitor (CDK4/6i) – ‘palbociclib’, which has shown promising results in breast cancer.

They found that talazoparib monotherapy did not inhibit tumor growth in mice injected with CRC cells. However, differential gene expression analyses revealed that this treatment activated cellular senescence signaling pathways. Additionally, talazoparib treatment led to the activation of the p53 signaling pathway, in turn enhancing the expression of p21 (known for its tumor suppressive roles). Further experiments revealed that talazoparib stabilizes p53 by enhancing its interaction with PARPi.

Notably, an increase in p21expression has been proven to sensitize cancer cells to CDK4/6i. The authors speculated that treatment with talazoparib could enhance the effects of CDK4/6i. Indeed, the combination of talazoparib and palbociclib significantly inhibited the proliferation of CRC cells. Moreover, the treatment was well‐tolerated in mice, and effectively inhibited their tumor growth.

Next, Wang et al. investigated the molecular mechanisms underlying the combined effects of talazoparib and palbociclib. They observed that this combination induced a senescence‐associated secretory phenotype, mediated by the activation of cGAS/STING signaling and type‐1 interferon responses. Moreover, it triggered innate immune responses and induced an anti‐tumor microenvironment, resulting in further suppression of tumor growth.

Considering the involvement of immune responses and T‐cell activation, the authors sought to assess the effect of suppressing PD‐L1 – an immune checkpoint inhibitor – in addition to the dual inhibition of PARP and CDK4/6. As expected, triple therapy significantly improved survival rates and tumor remission in mice.

Overall, these findings highlight a promising “one‐two punch” therapeutic strategy that combines the anti‐tumor and immunomodulatory effects of PARPi, CDK4/6i, and PD‐L1 inhibition, for the treatment of CRC.

https://onlinelibrary.wiley.com/doi/full/10.1111/cas.15961

## Collagen XVII regulates tumor growth in pancreatic cancer through interaction with the tumor microenvironment


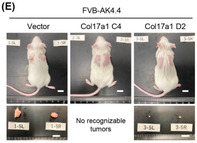


Pancreatic adenocarcinoma (PAAD) is one of the most refractory cancer types. The low treatment success rate is believed to be due in part to its characteristic tumor microenvironment. The tumor microenvironment—the surrounding environment of a tumor that includes immune cells and abundant cancer‐associated fibroblasts—plays an important role in the growth of tumor cells. Previous studies have suggested *COL17A1*, a gene encoding collagen XVII protein, as a potential diagnostic and prognostic biomarker for PAAD.

Expression of *COL17A1* is known to positively or negatively affect the survival of patients depending on the type of cancer. Whereas COL17A1 expression was found to promote cell invasion in colorectal cancer and squamous cell carcinoma, it suppressed cell migration and invasion in PAAD and invasive breast carcinoma. The mechanism underlying this complex role of COL17A1 in the progression of cancer is still unknown.

In this study, researchers examined the role and mechanism of action of COL17A1 in the development and progression of PAAD. They investigated the effect of expression of COL17A1 on growth of lab‐cultured pancreatic cancer cells and in tumor‐bearing mice.

They observed that expression of COL17A1 had no effect on the growth of lab‐cultured pancreatic cancer cells. However, when they transplanted those cells into mice, tumor growth of COL17A1‐expressing cells was promoted or suppressed depending on the genetic background of tumor cells. These observations indicate that genetic background of tumor cells influences the effect of COL17A1 on tumor growth. Furthermore, analysis of the tumor tissue from these mice showed that COL17A1 influences expression of genes involved in cell growth and immune cell responses in tumor environment.

Together, these findings suggest that COL17A1 either promotes or suppresses tumor growth depending on how the tumor cells interact with the tumor microenvironment.

https://onlinelibrary.wiley.com/doi/full/10.1111/cas.15952

## BPIFB1 promotes metastasis of hormone receptor‐positive breast cancer via inducing macrophage M2‐like polarization


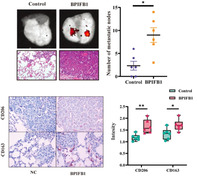


Breast cancer (BC), especially the hormone receptor‐positive subtype (HR+ BRCA), remains a major health challenge despite available diagnostic and treatment options. BC's poor prognosis is further exacerbated by metastasis (spread of cancer cells to other parts of the body) through the lymph nodes. In this regard, understanding the molecular mechanisms of metastasis is crucial for developing better treatments.

The BPIFB1 protein is believed to play a role in the development of different types of cancer, including BC. While it has been found to be more active in cancerous breast tissue, researchers are still trying to understand exactly how it contributes to BC.

Recently, a research by Hu et al. led to a fascinating discovery. It appears that BPIFB1 has the ability to promote the polarization of macrophages toward a specific M2‐like phenotype. When these immune cells encounter BPIFB1, they undergo a transformation in their behavior, effectively becoming allies of BC. They assist in its growth and spread within the body.

By analyzing gene expression data, the authors found that BPIFB1 expression was higher in patients with BC, especially those with HR+ BRCA tumors that had spread to the lymph nodes. This heightened BPIFB1 expression was associated with a poor prognosis and lower overall survival rates for these patients. The authors also looked at the genes involved, and found connections to both the estrogen signaling pathway and various immune‐related pathways.

To validate their discoveries, the authors conducted experiments to understand how BPIFB1 expression affects the activation of M2‐like macrophages and their involvement in cancer metastasis. The results were revealing: BPIFB1 promoted the polarization of the M2 subtype when BC cells were cultured alongside THP1 cells (human monocyte macrophages) or RAW264.7 cells (mouse macrophages). Additionally, increasing the levels of BPIFB1 led to larger tumors in experiments simulating lung metastasis.

These findings provide valuable insights into how BPIFB1 may be influencing BC development and progression. The research offers crucial insights into the role of BPIFB1 in BC, especially HR+ BRCA. By understanding how BPIFB1 influences immune cells and contributes to cancer can make it less life‐threatening and more manageable.

This study is thus a significant contribution to the field, providing hope and potential solutions for patients with BC.

https://onlinelibrary.wiley.com/doi/full/10.1111/cas.15957

